# Preservation of H_2_ production activity in nanoporous latex coatings of *Rhodopseudomonas palustris* CGA009 during dry storage at ambient temperatures

**DOI:** 10.1111/1751-7915.12032

**Published:** 2013-07-01

**Authors:** M Piskorska, T Soule, J L Gosse, C Milliken, M C Flickinger, G W Smith, C M Yeager

**Affiliations:** 1 University of South Carolina, AikenAiken, SC, 29801, USA; 2 Savannah River National LaboratoryAiken, SC, 29808, USA; 3 North Carolina State UniversityRaleigh, NC, 27695, USA; † Indiana University-Purdue UniversityFort Wayne, IN, 46805, USA; ‡ BioCee, Inc.Minneapolis, MN, 55414, USA; § Bioscience Division, Los Alamos National LaboratoryLos Alamos, NM, 87545, USA

## Abstract

To assess the applicability of latex cell coatings as an ‘off-the-shelf’ biocatalyst, the effect of osmoprotectants, temperature, humidity and O_2_ on preservation of H_2_ production in *Rhodopseudomonas palustris* coatings was evaluated. Immediately following latex coating coalescence (24 h) and for up to 2 weeks of dry storage, rehydrated coatings containing different osmoprotectants displayed similar rates of H_2_ production. Beyond 2 weeks of storage, sorbitol-treated coatings lost all H_2_ production activity, whereas considerable H_2_ production was still detected in sucrose- and trehalose-stabilized coatings. The relative humidity level at which the coatings were stored had a significant impact on the recovery and subsequent rates of H_2_ production. After 4 weeks storage under air at 60% humidity, coatings produced only trace amounts of H_2_ (0–0.1% headspace accumulation), whereas those stored at < 5% humidity retained 27–53% of their H_2_ production activity after 8 weeks of storage. When stored in argon at < 5% humidity and room temperature, *R. palustris* coatings retained full H_2_ production activity for 3 months, implicating oxidative damage as a key factor limiting coating storage. Overall, the results demonstrate that biocatalytic latex coatings are an attractive cell immobilization platform for preservation of bioactivity in the dry state.

## Introduction

The encapsulation of living cells to create living hybrid materials for use as biocatalysts, biosensors and bioremediation shows tremendous promise and has been the subject of extensive research for decades (Scott, 1987; Bjerketorp *et al*., 2006; Wang *et al*., 2010; Michelini and Roda, 2012). One of the fundamental challenges facing the successful commercialization of immobilized cell devices is how to preserve biological activity while retaining functionality and affordability (Bjerketorp *et al*., 2006; Wang *et al*., 2010; Michelini and Roda, 2012). Storage conditions that minimize metabolic activity, such as low temperatures or low relative humidity (i.e. freeze-dried, −80°C, under vacuum, etc.), are widely used and have a long history for microbial preservation; however, some of these require continuous cold storage and very little attention has been paid to the application of non-refrigerated conditions for cell preservation in modern immobilization matrices such as sol-gels or latex (Bjerketorp *et al*., 2006; Morgan *et al*., 2006; Tessema *et al*., 2006; Soltmann and Böttcher, 2008; Kuppardt *et al*., 2009). Furthermore, almost all immobilization matrices require liquid immersion or a humid atmosphere in order to maintain bioactivity of the immobilized cells (Bjerketorp *et al*., 2006; Michelini and Roda, 2012; Pannier *et al*., 2012). A notable exception has been the development of freeze-gelation techniques for biologically active biocers (biological ceramic composites) (Koch *et al*., 2007; Soltmann and Böttcher, 2008; Pannier *et al*., 2012).

Adhesive latex binders are a stable, non-toxic, nanoporous matrix that have advantages compared with other immobilization matrices, such as alginate and sol-gel, because it is adhesive, economical (produced in very large quantities at low cost for the water borne coating industry), does not collapse upon drying, and can be used to immobilize very high concentrations of cells (Flickinger *et al*., 2007). Experimental biocatalytic latex coatings have been designed for a variety of applications including: mercury detection, microbial fuel cell technology, high intensity chiral oxidations, and biocatalysis by thermophiles (Lyngberg *et al*., 1999,2005; Fidaleo *et al*., 2006; Srikanth *et al*., 2008). Adhesive latex-based coatings are also ideal for photosynthetic H_2_ production because they provide a high surface area to volume ratio for incident light, efficient rates of gas diffusion, and can significantly increase cell longevity by protecting the microorganisms from mechanical degradation and contamination (Lyngberg *et al*., 2001; Flickinger *et al*., 2007). Indeed, latex coatings have been successfully developed for H_2_ production using the purple non-sulfur phototroph, *Rhodopseudomonas palustris*, with stable rates of H_2_ production (2.08 ± 0.01 mmol H_2_ m^−2^ day^−1^) observed for > 4000 h when cell/latex coatings were hydrated in liquid medium that was periodically refreshed (Gosse *et al*., 2007,2010).

*Rhodopseudomonas palustris* produces H_2_ via nitrogenase as an obligate product of enzyme turnover (N_2_ + 8e^−^ + 8H^+^ + 16ATP → 2NH_3_ + H_2_ +16ADP + 16Pi), and in the absence of N_2_, nitrogenase acts as an ATP-powered hydrogenase where all electrons shuttled to the nitrogenase are available for H_2_ production (2H^+^ + 2e^−^ + 4ATP → H_2_ + 4ADP + 4Pi) (McKinlay and Harwood, 2010). *Rhodopseudomonas palustris* is an excellent candidate for biological H_2_ production because it can grow phototrophically and utilize a broad range of organic molecules as a source of electrons to support nitrogenase activity (i.e. organic acids, lignin monomers and other agricultural and food wastes) (McKinlay and Harwood, 2010). Indeed, it was recently demonstrated that an inorganic electron source, thiosulfate, can also support nitrogenase activity in this versatile microorganism (Huang *et al*., 2010). H_2_ production efficiency is constrained in growing cultures of *R. palustris* as most electrons are routed for biosynthesis rather than to nitrogenase. However, *R. palustris* can generate up to 80% of the theoretical H_2_ yields when sustained in a non-growing state (Akkerman *et al*., 2002). Importantly, cells of *R. palustris* can remain active in a non-growing state through light-driven cyclic photophosphorylation, which maintains membrane H^+^ gradients and intracellular ATP levels (Melnicki *et al*., 2008).

Preservation methods to ensure that latex-embedded cells can undergo long-term storage while maintaining activity have not been investigated. Coating preparation involves a controlled coalescence/drying step and, if needed, a subsequent short-term storage period, both affected by temperature and usually carried out at 60% relative humidity (Gosse *et al*., 2007). While this strategy has proven successful when coatings are rehydrated for use within several days of preparation, for industrial ‘off-the-shelf’ applications of microbial coatings, economics and practicality necessitate that coatings be stored in a dry state with stable reactivity over a much longer time frame (months–years). Storage conditions (temperature, humidity, O_2_ tension, illumination or dark, etc.) must be defined to enable long-term dry storage of latex-embedded microbial cells, preferably without the requirement for refrigeration.

Cells in coatings are subjected to desiccation stress, including osmotic shock, as the latex emulsion dries forming particle–particle coalescence and adhesion to the substrate resulting in cell immobilization. To moderate this stress, cell/latex formulations have been supplemented with sucrose, glycerol and other osmotic stabilizers (Yoo and Lee, 1993; Leslie *et al*., 1995; Lyngberg *et al*., 2001). Additionally, glycerol and carbohydrates serve to arrest polymer particle coalescence during film formation and thus increase nanoporosity within the coatings. Previous studies have demonstrated that these additives are required to maintain optimal levels of cell viability and activity in coatings upon rehydration 24–48 h after formation (Flickinger *et al*., 2007; Gosse *et al*., 2007). However, the potential osmoprotective role of these compounds over long-term periods of dry storage has not been examined in bioactive latex coatings.

In order to advance the development of latex-embedded *R. palustris* cells as lightweight, portable catalysts for H_2_ production, we examined the effectiveness of (i) osmotic stabilizers such as glycerol, sucrose, trehalose and sorbitol and (ii) modifications to temperature, humidity and atmospheric O_2_ concentration during the critical film formation coalescence and storage periods to extend the shelf life of *R. palustris* coatings. The goal of our work here was to identify conditions, including the addition of trehalose, sorbitol or succinate, that preserve the H_2_ production capacity of dry *R. palustris* coatings over month-long storage periods at room temperature. Here, we establish that biocatalytic latex coatings of *R. palustris* can be stored in a dry state at room temperature for up to 3 months, while retaining their original H_2_ production activity. To our knowledge, this is the first report of a method to preserve photobiological activity of embedded cells during dry storage.

## Results

### Effect of osmotic stabilizers on H_2_ production by *R. palustris* latex coatings

**Figure 1:**
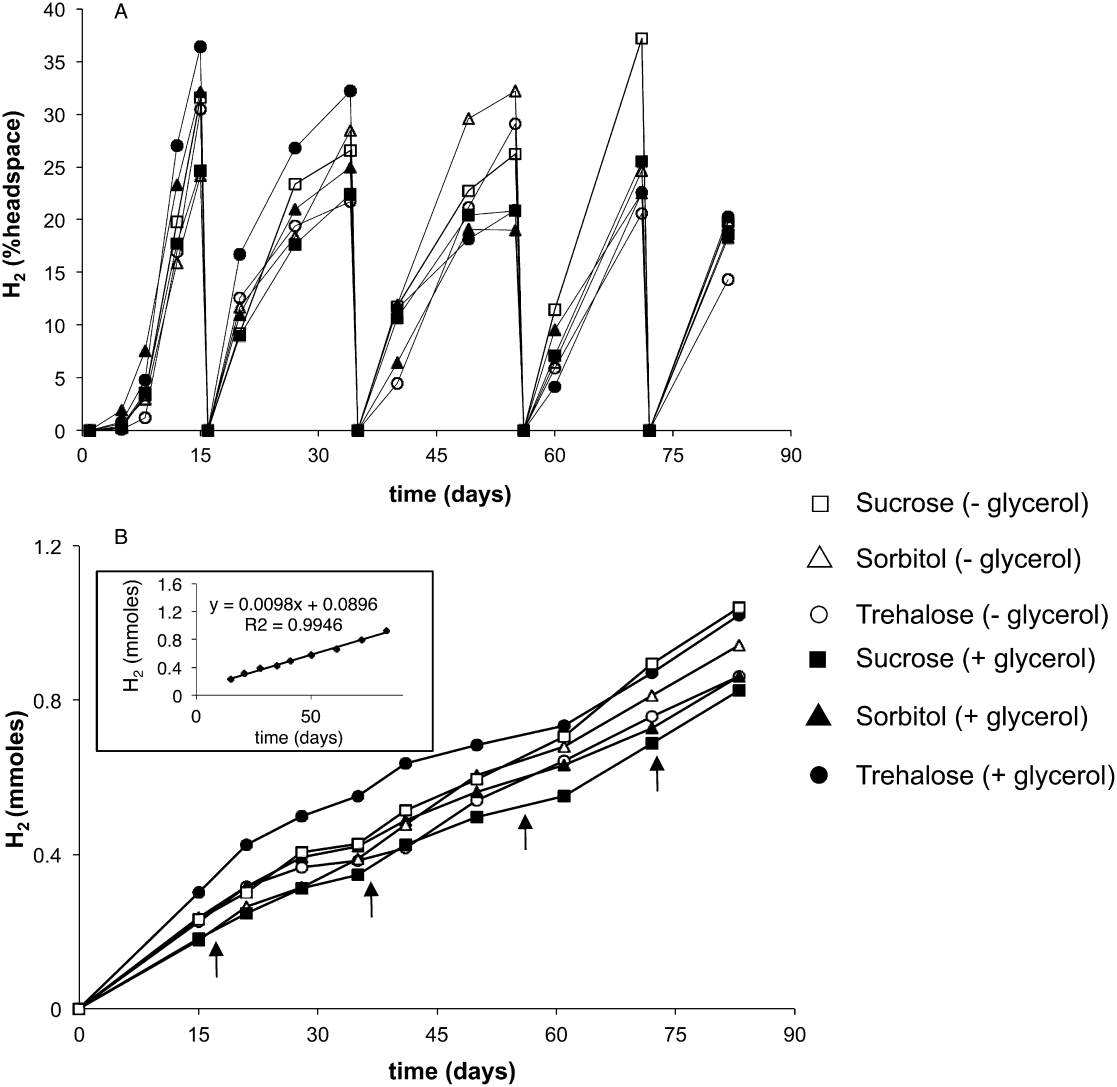
H_2_ production by *R. palustris* latex coatings containing different osmotic stabilizers (sucrose, sorbitol or trehalose; with or without glycerol). Freshly prepared *R. palustris* coatings were stored at 22°C under 60% humidity for 2 days prior to hydration in PM(NF) medium and initiation of the H_2_ production assays.A. H_2_ production is presented as per cent accumulation in the headspace over time where the headspace of each is tube is refreshed periodically with argon upon medium replacement (∼ every 2 weeks).B. Cumulative H_2_ production from the same coatings with arrows representing each medium replacement/headspace-flushing event. The inset in (B) shows the average rate of H_2_ production of all coatings, 0.82 mmoles H_2 _m^−2 ^h^−1^.Symbols represent average values obtained from duplicate experiments.

The relative efficacy of three osmotic stabilizers for maintaining H_2_ production in *R. palustris* coatings following the initial dehydration period was examined. Latex formulations were supplemented with sucrose, sorbitol or trehalose, with or without glycerol, and stored for 2 days at 22°C with 60% humidity. Regardless of the osmolyte combination tested, coatings of *R. palustris* maintained H_2_ production for at least 85 days and after multiple media replacements (Fig. 1A). As observed previously (Gosse *et al*., 2007,2010), H_2_ production rates were highest immediately after hydration, and lower, but stable, after subsequent medium replacement/headspace flushing events (0.82 mmol H_2_ m^−2 ^h^−1^ over ∼ 80 days; Fig. 1B inset). Rates of H_2_ production were similar among the stabilizer formulations tested (Fig. 1B).

### Coalescence and storage temperature of *R. palustris* coatings and H_2_ production

**Figure 2:**
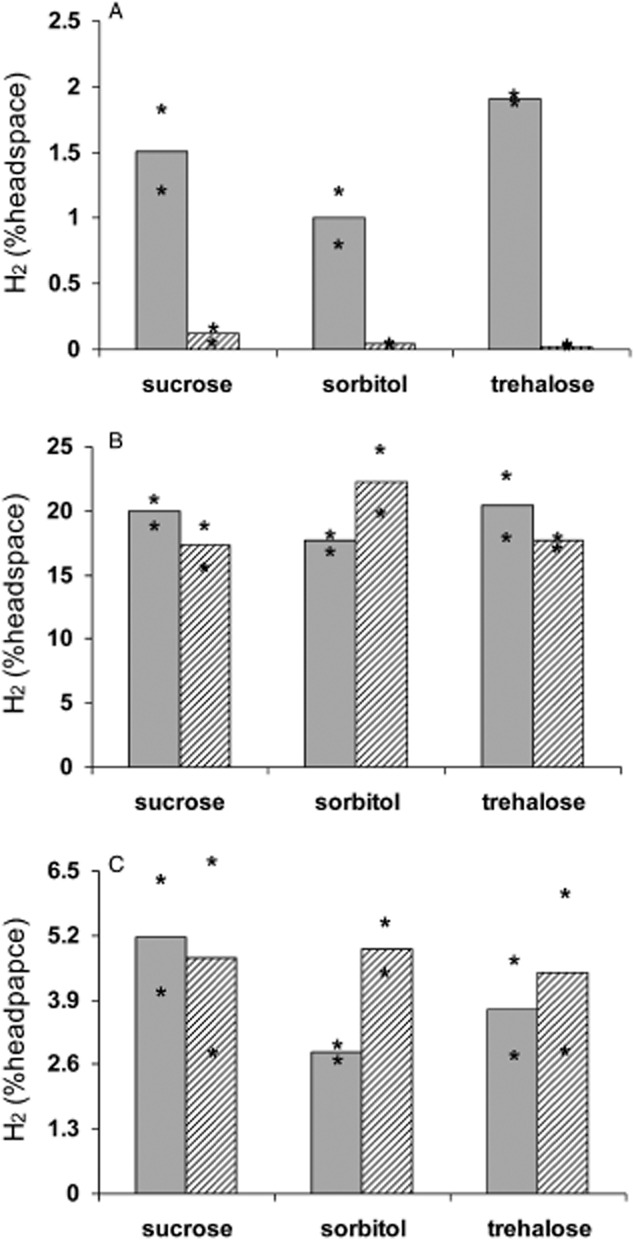
Hydrogen production by *R. palustris* cells embedded in latex containing glycerol along with sucrose, sorbitol or trehalose and prepared/stored at 4°C (grey bars) or 22°C (striped bars) under 60% humidity for 7 days. Hydrogen production is presented as the per cent H_2_ in the headspace (A) 5 days post hydration, (B) 19 days post hydration and (C) 3 days after replacing the medium and flushing the headspace on day 19. Bars are average values of duplicate coatings and stars delimit the range of values.

Here, we tested the hypothesis that coatings coalesced and stored at cold temperatures would maintain higher H_2_ production levels following hydration. *Rhodopseudomonas palustris* latex coatings stabilized with glycerol and sucrose, sorbitol or trehalose were dried and stored at either 22°C or 4°C (7 days, dark, 60% humidity). Initially (up to 5 days post hydration), H_2_ production was much higher (6- to 10-fold) in coatings prepared and stored at 4°C than those treated at 22°C (Fig. 2A). However, H_2_ accumulation was similar between all treatments following 19 days of incubation (Fig. 2B), and 3 days after a subsequent medium replacement/flushing event (Fig. 2C).

### H_2_ production by *R. palustris* coatings after long-term storage

To assess the ability of latex-embedded *R. palustris* cells to maintain activity over long-term storage, coatings containing different osmotic stabilizers were stored up to 4 weeks at 60% humidity then assayed for H_2_ production (Table 1). Coatings stored for 14 days exhibited comparable H_2_ yields to fresh coatings that were rehydrated and assayed < 24 h after coalescence. After 28 days of storage, two of three sucrose coatings, one of three trehalose coatings and all three sorbitol coatings failed to produce H_2_. The single active sucrose coating exhibited decreased H_2_ production yields (9.1% headspace H_2_) compared with fresh coatings (29% H_2_ headspace), whereas the two active trehalose coatings exhibited modest losses of H_2_ production activity compared with fresh coatings (25% and 23% H_2_ headspace). None of the coatings generated H_2_ after 56 days of storage at 60% humidity.

**Table 1:** Hydrogen production by *R. palustris* coatings after storage for up to 56 days at 22°C and 60% humidity

Storage period (days)	H_2_ accumulation (% headspace)^a^		
	Sucrose^b^	Trehalose^b^	Sorbitol^b^
< 1	29.0 ± 0.5^c^	30.4 ± 6.4	31.9 ± 8.9
14	25.2 ± 1.9	21.3 ± 0.4	32.2 ± 12.6
28	3.3 ± 5.6	16.8 ± 14.5	0 ± 0
56	0 ± 0	0 ± 0	0 ± 0

a H_2_ accumulation was measured 20 days after coatings had been rehydrated in PM(NF) medium in closed vessels containing an argon atmosphere.

b Coatings contained glycerol plus the indicated osmolyte stabilizers.

c Values are averages from three replicate strips ± SD.

To determine if *R. palustris* coatings could maintain greater H_2_ production capability when stored under conditions of low relative humidity, coatings stabilized with glycerol and either sucrose, trehalose or sorbitol were stored for 28 days at < 5% or 60% relative humidity. Sucrose and trehalose coatings retained 67% and 59% of their respective H_2_ production activity when stored at < 5% humidity over this time period. Importantly, all of the sucrose and trehalose coating replicates (three of each) retained H_2_ production activity when stored at < 5% humidity, and there was little strip to strip coating (technical replicate) variability (data not shown). Sorbitol coatings did not produce H_2_ after 28 days of storage, either at 60% or at < 5% humidity.

Additional experiments were conducted, with sucrose and trehalose as stabilizers (+ glycerol), to more fully assess the effects of storage humidity on H_2_ production by *R. palustris* coatings. As in our previous experiment, coatings stored for 28 days or longer exhibited very little or no H_2_ production activity when stored at 60% humidity and 22°C (Table 2). In contrast, when stored at < 5% humidity the sucrose and trehalose coatings retained significant activity, even after 56 days of storage (27% and 53% of the original activity respectively). When stored at < 5% humidity, each coating replicate (three of each) exhibited activity and the strip to strip variability was relatively small. It is also important to note that when stored at < 5% humidity, very little activity was lost from the coatings between 4 and 8 weeks of storage. Coatings initially dried under an atmosphere of < 5% relative humidity (48 h) then placed at 60% humidity behaved similarly to coatings that were coalesced *and* stored at 60% humidity – with complete (or nearly complete) loss of H_2_ production activity after 28 days of storage (Table 2).

**Table 2:** Hydrogen production by *R. palustris* coatings after storage for up to 56 days at 22°C and < 5% or 60% humidity

Storage period (days)	H_2_ accumulation (% headspace)^a^					
	Sucrose^b^			Trehalose^b^		
	< 5%	60%	60%^c^	< 5%	60%	60%^c^
7	51.1 ± 5.5^d^	9.4 ± 0.9	9.0 ± 2.4	35.2 ± 1.0	14.1 ± 3.4	9.3 ± 1.7
28	15.7 ± 0.7	0 ± 0	0 ± 0	20.2 ± 0.8	0.1 ± 0.1	0 ± 0
56	13.8 ± 4.4	0 ± 0	0 ± 0	18.5 ± 0.6	0 ± 0	0 ± 0

a H_2_ accumulation in argon measured 20 days post rehydration in PM(NF).

b Coatings contained glycerol plus the indicated osmolyte stabilizer.

c Coatings dried for 48 h at < 5% humidity, then stored at 60% humidity.

d Values are averages from three replicate strips ± SD.

### Respiratory activity of latex-embedded *R. palustris* after long-term storage

**Figure 3:**
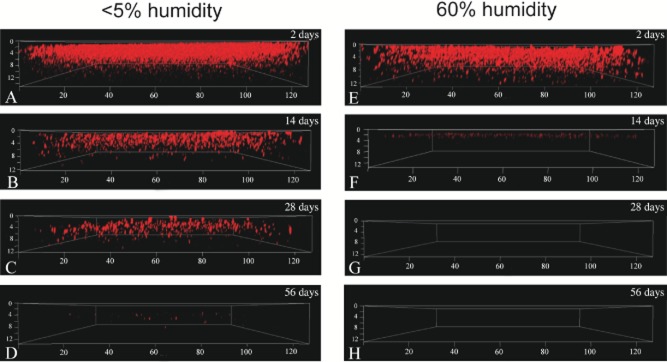
Three-dimensional views of *R. palustris* coatings (+ sucrose/glycerol) stained with CTC (the axes distances are shown in μm). Coatings were stored at room temperature in the dark for 2, 14, 28 or 56 days at < 5% humidity (A–D) or 60% humidity (E–H).

*Rhodopseudomonas palustris* cells embedded in latex were examined for anaerobic respiratory activity after various storage periods using 5-cyano-2, 3-ditolyl tetrazolium chloride (CTC). *Rhodopseudomonas palustris* coatings stabilized with sucrose (+ glycerol) that had been stored in the dark for 2, 14, 28 or 56 days under either < 5% humidity or 60% humidity at 22°C were rehydrated anaerobically in PM(NF) medium and stained with CTC. CTC-stained coatings were examined with a confocal laser-scanning microscope to evaluate cell anaerobic respiratory activity of the embedded *R. palustris* cells (Fig. 3). Image analysis revealed dense, actively respiring cells through the z-plane when the coatings were stored at < 5% humidity for 2 days, while a less dense population was observed in coatings stored at 60% humidity (1.3- to 3.4-fold less CTC-stained cells based on image analysis; Table 3). Longer storage times resulted in a marked decrease in cell respiratory activity, which was strongly dependent on the storage humidity. Coatings stored at 60% humidity contained very few CTC-stained cells after 14 days of storage, whereas coatings stored at < 5% humidity still contained an appreciable number of active cells (on average, ∼ 15-fold more than the coatings stored at 60% humidity). CTC-stained cells were not detected in coatings stored at 60% humidity after 56 days storage, and < 1% of the number of CTC-stained cells enumerated in the 2-day-old coatings remained in the coatings stored at < 5% humidity (Table 3).

**Table 3:** Number of CTC stained spots, representing actively respiring cells of *R. palustris*, detected at three depths in coatings (+ sucrose/glycerol) following storage at either < 5% or 60% relative humidity

Coating depth (μm)	< 5% Humidity Storage period (days)				60% Humidity Storage period (days)			
	2	14	28	56	2	14	28	56
5	2217 ± 26	217 ± 6	145 ± 6	17 ± 4	654 ± 10	13 ± 5	5 ± 2	0
8	2386 ± 9	103 ± 4	94 ± 8	5 ± 2	841 ± 7	8 ± 1	21 ± 3	0
10	1023 ± 15	84 ± 4	92 ± 8	3 ± 1	837 ± 6	0	10 ± 3	0

Data are presented as the number of fluorescent spots detected in each plane of analysis. Values represent triplicate measurements (± SD), where fluorescent spots were enumerated for three randomly selected fields of view for each plane of analysis (coating depth).

**Figure 4:**
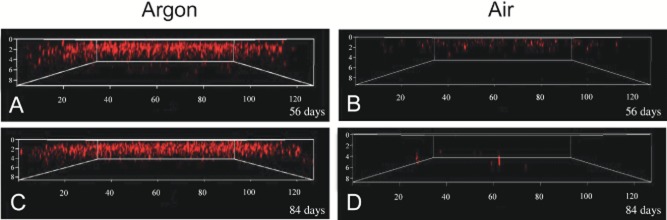
Three-dimensional views of *R. palustris* coatings (+ sucrose/glycerol) stained with CTC (the axes distances are shown in μm). Coatings were stored at room temperature in the dark at < 5% humidity under argon (A, C) or air (B, D) for 56 or 84 days. It is important to note that although the images for Figs 3 and 4 were taken identically, the brightness and contrast of the images in Fig. 4 were adjusted to enable better visualization; thus, comparisons of fluorescent intensity between Figs 3 and 4 is not appropriate. However, each of the Fig. 4 images was treated identically so that comparisons between these images are still valid.

Interestingly, the highest density of CTC-stained cells observed in the 2-day-old coatings was located in the upper portion of the coating, but after long-term storage, CTC-stained cells tended to cluster further into the coating interior (Fig. 3). These results led us to hypothesize that cellular damage incurred in coatings over long-term storage periods was related to oxygen exposure. Thus, sucrose or trehalose (+ glycerol) coatings were stored under argon or air for a period of 8–12 weeks at room temperature, and then assayed for H_2_ production and CTC response. After 8 weeks of storage at < 5% humidity, *R. palustris* coatings stored in an argon atmosphere exhibited two- to fourfold higher H_2_ production activity than coatings stored in air (Table 4). After 12 weeks of storage H_2_ accumulation was markedly higher (7- to 17-fold) in coatings stored in argon versus air. Notably, the H_2_ production activity measured in *R. palustris* coatings that had been stored in argon for 12 weeks was equal to or greater than the activity observed in freshly prepared coatings (Tables 1, 2 and 4). In accordance with these results, numerous CTC-stained cells were observed in coatings stored at < 5% humidity in argon following an 8- to 12-week storage period (Fig. 4), whereas few actively respiring cells were detected in coatings that were treated similarly but stored under air. Additionally, the CTC-stained cells did not appear to cluster preferentially in the interior of the coating after storage in argon. These results demonstrate that H_2_ producing coatings of *R. palustris* can be stored dry, at room temperature, and maintain full activity for up to 3 months.

**Table 4:** Hydrogen production by *R. palustris* coatings after storage for 8–12 weeks at < 5% relative humidity under air or argon

Storage period (weeks)	H_2_ accumulation (% headspace)^a^			
	Sucrose (+ glycerol)		Trehalose (+ glycerol)	
	Air	Argon	Air	Argon
8	12.7 ± 0.3^c^	48.0 ± 7.8	9.8 ± 1.7	31.5 ± 4.2
8 (2nd flush)^b^	15.5 ± 2.3	36.3 ± 1.8	15.8 ± 2.0	30.3 ± 1.5
12	9.5 ± 1.0	69.1 ± 8.1	3.1 ± 1.0	52.3 ± 4.0

a H_2_ accumulation in argon 10 days post rehydration in PM(NF) medium.

b H_2_ accumulation in argon (10-day incubation) following the initial H_2_ production period (20 days) and one flushing/medium refresh event.

c Values are averages from three replicate strips ± SD.

## Discussion

An important technical hurdle that must be addressed before biocatalytic latex coatings can be used as ‘off-the-shelf’ catalysts for H_2_ production, or other applications, is that of maintaining bioactivity stability during long-term storage. In this study, latex-embedded cells of *R. palustris* were stored in a dry state at room temperature for up to 3 months while maintaining their original H_2_ production activity. Successful preservation of cell activity required the addition of select osmotic stabilizers, i.e. sucrose or trehalose, to the coating mixture, low relative humidity (< 5%) and anaerobic conditions during storage. It is important to note that we did not determine the dry storage lifetime of *R. palustris* coatings in this study. Yet, based on the observation that H_2_ production activity remained relatively stable over the final two sampling periods of this study (8 and 12 weeks), it seems likely that activity could be preserved in *R. palustris* coatings for greater periods of time.

*Rhodopseudomonas palustris* coatings dried and stored at 4°C produced greater amounts of H_2_ than those treated similarly at 22°C − in the days immediately following hydration. Over time (10–20 days post hydration), however, H_2_ production activity was similar between *R. palustris* coatings that had been stored at 4°C and 22°C. These results could signify that coatings stored at 4°C are capable of initiating H_2_ production activity quicker than those stored at 22°C, but that latex-embedded cells stored at 22°C can eventually recover full activity after periods of short term (1 week) storage. Alternatively, diminished particle coalescence at 4°C could lead to greater rates of acetate diffusion through the latex matrix upon hydration, thus greater H_2_ yields for *R. palustris* coatings prepared and stored at this temperature. As the coatings age under hydration, the sugars tend to leach into the medium and particle coalescence resumes, thus the permeability decreases and acetate accessibility would become more uniform for both treatment temperatures (Lyngberg *et al*., 2001).

The primary goal of this particular experiment was to determine if decreased temperatures during the film formation and polymer particle coalescence process and initial dry storage period would have an impact on H_2_ production activity following hydration of the coatings − not to determine if low temperatures could be used as a long-term storage strategy. In our view, storage at room temperature is more compatible with many of the shipping, storage and handling constraints that would make latex embedded cells attractive as a lightweight, off-the-shelf biocatalyst or biosensor technology. The observation that *R. palustris* coatings exhibit a shorter lag in hydrogen production response time when prepared and stored at 4°C has implications for applications, such as biosensors, where a ready-to-use system exhibiting immediate activity is essential.

Freeze-drying and controlled drying without freezing are the methods of choice used by industry to preserve microbial cells, and while these techniques are primarily applied to cell suspensions or pastes, they have also been investigated as techniques to preserve immobilized cells. For example, bacterial sensor cells targeting molecules as diverse as *N-*acylhomoserine or arsenite/arsenate have been air dried onto filter paper, lyophilized, and stored at 4°C for 3 months or 30°C for 2 months without appreciable loss of reporter activity (Stocker *et al*., 2008; Struss *et al*., 2010). Freeze drying has also been applied to cells immobilized in sol-gels (Tessema *et al*., 2006; Meunier *et al*., 2010). However, freeze-dried cells must not be exposed to moisture and, despite high initial cell suspensions (greater than 10^8^), survival rates of the original cell population can be as low as 0.1% (Bozoglu *et al*., 1987; Miyamoto-Shinohara *et al*., 2000,2008). These cell viability rates may be acceptable for propagation of the strain but are incompatible with biocatalytic latex coatings that are engineered for a high reactivity per unit of surface area (high intensity) and where cell growth is limited. Nonetheless, because our results demonstrate that latex-embedded cells of *R. palustris* retain considerable activity when stored under low relative humidity and respond quicker when prepared at lower temperatures, the applicability of freeze drying biocatalytic latex coatings for long-term storage (> 1 year) should be evaluated [latex embedded *R. palustris* cells were previously shown to maintain activity after storage for 1 year at −80°C (Gosse *et al*., 2010)].

Regarding osmotic stabilizers, rates of H_2_ production by *R. palustris* coatings were quite similar for each of the formulations tested (sucrose, sorbitol and trehalose ± glycerol) when the strips were fresh or stored for ≤ 2 weeks. Coatings prepared with either trehalose or sucrose retained 31–67% of their H_2_ production activity through 28 days under low humidity and 27–53% activity through 56 days of storage. In contrast, sorbitol-stabilized coatings were inactive regardless of the relative humidity levels beyond 2-week storage time. Although we did not investigate the underlying factors responsible for the differences in stabilizer performance, other studies have concluded that the efficacy of sorbitol as an osmoprotectant is quite variable (de Valdez *et al*., 1983; Carvalho *et al*., 2003), resulting in a greater emphasis on sucrose and trehalose as stabilizers (Leslie *et al*., 1995; Lyngberg *et al*., 2001).

The two elements found to be critical for preserving H_2_ production activity in *R. palustris* coatings were (i) low relative humidity and (ii) low O_2_ levels during the storage period. Since slower drying rates allow for greater polymer particle mobility, coatings that are dried at 60% humidity have greater permeability than those dried at lower humidity (Lyngberg *et al*., 1999) resulting in greater porosity for gas and nutrient diffusion (Sperry *et al*., 1994; Ma *et al*., 2005). Yet, in this study we detected essentially no H_2_ production activity in *R. palustris* coatings stored at 60% humidity under air after 1 month. In addition to this loss of H_2_ production activity (Table 2) and general cell respiratory activity (Fig. 3), visual inspection of the *R. palustris* coatings also revealed differences between coatings stored under low or high humidity (data not shown). The characteristic red-purplish pigmentation of *R. palustris* dulls to a light red/orange colour over time during dry storage at 60% humidity, suggesting loss of light-harvesting bacteriochlorophyll or carotenoids. Coatings stored at < 5% humidity were much more resilient to pigmentation loss. We suspect that long-term storage at 60% humidity may provide the latex-embedded cells enough moisture to support low levels of metabolic activity, which could result in energy depletion over time or accumulation of metabolites in the pore space adjacent to cells that could become either toxic or exert osmotic stress on the embedded cells.

Oxidative stress is well known as a cause of cell damage and death during long-term storage (Dimmick *et al*., 1961; Meng *et al*., 2008). Oxidative damage to DNA, proteins, and particularly the cell membrane, has been implicated as a major contributor to the viability losses often observed when dried microorganisms are exposed to air for extended periods (Marshall *et al*., 1974; Israeli *et al*., 1975; Teixeira *et al*., 1996; Vriezen *et al*., 2007; Scherber *et al*., 2009). Water-deficient cells are unable to actively neutralize or excrete oxygen radicals or repair oxidative damage, thus cellular injury would inevitably and slowly accumulate until a threshold is reached beyond which cell recovery is improbable. In this study, we provide two lines of evidence that oxidative stress is an impediment to the long-term storage of *R. palustris* coatings. First, coatings stored under an argon atmosphere retained > 10 times greater H_2_ production activity than those stored under air, even under conditions of low relative humidity where metabolic activity should be minimal. Second, as storage time elapsed under air, respiratory activity (assayed under anaerobic conditions) in rehydrated coatings was detected in *R. palustris* cells that tended to be clustered in the interior of the latex coating (Fig. 3), where O_2_ exposure during storage would be expected to be less than at the edges. In contrast, active cells were detected relatively evenly throughout the vertical profile of coatings that had been stored under argon (Fig. 4).

The enzyme responsible for H_2_ production in *R. palustris*, nitrogenase, is highly sensitive to oxygen (Gallon, 1992); therefore, exposure to O_2_ during long-term storage could result in longer lag times associated with H_2_ production (time required for repair or *de novo* synthesis of nitrogenase). Longer lag periods were noted for *R. palustris* coatings stored under air versus argon (data not shown), which could account for the large difference in H_2_ yields exhibited by coatings stored under these two conditions immediately following hydration (Table 4). Nonetheless, because coatings stored under argon continued to produce H_2_ at rates > 10× that of air-stored coatings up to 40 days after rehydration and after medium replacement, nitrogenase inactivation is almost certainly not the only damage incurred by latex-embedded *R. palustris* cells upon long-term storage in the presence of air. Accordingly, the CTC staining experiments revealed that general cell respiratory activity was compromised to a much greater extent in latex-embedded *R. palustris* stored in air.

Overall, this study demonstrates that biolatex coatings have great potential as an ‘off-the-shelf’ catalyst, considering that, with very little effort towards optimization, consistent retention of *R. palustris* activity was achieved following dry storage of coatings for at least 3 months at room temperature. If successfully applied to other microorganisms, the long-term storage properties of biocatalytic latex coatings would make it an attractive technology for a myriad of applications. For example, cell immobilization technologies applied to bioconversion, alternative fuel production, bioremediation, solar energy trapping, and food processing could benefit from long-term storage at room temperature using the methods described herein (Junter and Jouenne, 2004).

## Experimental procedures

### Bacterial strain, media, growth conditions and latex characteristics

*Rhodopseudomonas palustris* CGA009 was kindly provided by Dr Caroline Harwood, University of Washington. This strain produces hydrogen via three isozymes of the nitrogenase protein in an anaerobic environment at higher yields than the wild type due to an inactive uptake hydrogenase caused by a spontaneous frameshift mutation in the hydrogen sensor protein, *hupV* (Oda *et al*., 2005; Rey *et al*., 2006). *Rhodopseudomonas palustris* was cultured anaerobically in nitrogen fixing photosynthetic medium, PM(NF), supplemented with 20 mM acetate (unless otherwise noted, PM(NF) used throughout this study contained 20 mM acetate) in sealed glass serum bottles under a N_2_ atmosphere (Gosse *et al*., 2007). *Rhodopseudomonas palustris* was incubated statically under constant illumination at 60 μE with 60 W incandescent light bulbs at 31°C.

Latecies KAK4391 and Rhoplex™ SF012 (Rohm and Hass, Philadelphia, PA) latex formulations, both adjusted to pH 7.0, were used for this study. Latex KAK3941 is a vinyl acetate-*co*-acrylate that does not include biocide or hydroxyethylcellulose surface grafting, has a glass transition temperature (Tg) of 8.1°C, an average particle size of 280 nm, and a per cent solids of 52.5%. Rhoplex SF012 is a commercially available, acrylic *co*-polymer binder without biocide containing a solids content of 43.5%.

### Preparation of *R. palustris* latex coatings (Supplementary Fig. S1)

*Rhodopseudomonas palustris* cells were harvested in early stationary phase (OD_660_ ∼ 0.8) by centrifugation at 7600 *g* for 15 min at room temperature. Cell pellets were suspended in 50 ml of PM(NF) medium without acetate and transferred to pre-weighed 50 ml Falcon tubes. After centrifugation (as above), the supernatant was removed and the wet weight of the cell pellet was determined. Prior to latex addition, the bacterial cell paste was first mixed with the indicated amount of glycerol and/or sucrose, sorbitol or trehalose. The latex emulsions for coatings were prepared based on the formulation ratio of 1.2 g wet cell weight, 350 μl of 1.7 M sucrose, sorbitol or trehalose, 150 μl of 100% glycerol (exceptions are noted) and 1 ml of latex. The two initial experiments (Figs 1 and 2) were conducted using KAK4391 latex; however, due to a discontinuation of this product by the manufacturer, the remaining studies were performed with Rhoplex emulsion SF012. Importantly, when using the SF012 latex, the polyester template was first cleansed with a small amount of 1 M HCl to minimize hydrophobic tension in the formulation upon spreading.

*Rhodopseudomonas palustris* latex coatings were prepared as strips using a template design consisting of a glass support, a 125-μm-thick polyester sheet (DuPont Melinex 454, Tekra Corp, NJ), and an adhesive vinyl mask (84 μm thick, Con-Tact, Stamford, CT). The polyester sheet was pre-cut with parallel lines separated by 1 cm to define the width of each strip, and attached to a glass support covered with double-sided Scotch tape (strips perpendicular to the tape). An adhesive vinyl mask with a pre-cut rectangle (5 cm long to define the length of each strip; modified to 3.5 cm) was placed on top of the polyester so that its parallel pre-cut lines were in the centre of the mask opening. The width of the mask opening was determined by the number of polyester strips plus an additional 0.5 cm on each end [for template design details (Gosse and Flickinger, 2010)].

Coatings were prepared under aerobic conditions in an acrylic glove box (Plas By Labs, Lansing, MI) at 22°C and 60% humidity, unless otherwise noted. Humidity was measured using a dew point, wet-bulb humidity thermometer (Fisher Scientific, Pittsburgh, PA). The latex/cell formulation was transferred from a Falcon tube onto an assembled template mask where it was then spread across the top of the mask along the width of the polyester window with a pipette, minimizing bubble formation (see Supplementary Fig. S1 in *Supporting information*). A 26-wire wound Mayer rod (Paul N. Gardner, Pompano Beach, FL) was drawn by hand down the template mask in order to spread the formulation. The coatings were allowed to dry for 24 h (unless otherwise noted) in the glove box before removing the mask. Each individual polyester strip (1 × 5 cm, unless otherwise noted) ‘painted’ with embedded cells was then removed from the glass support and hydrated with 10 ml of PM(NF) medium in Balch tubes (Bellco Biotechnology, Vineland, NJ) (Gosse *et al*., 2007). Tubes were sealed with butyl septum stoppers, and flushed with argon for 30 min to produce an anaerobic environment for H_2_ production (16.5 ml of headspace). *Rhodopseudomonas palustris* coatings in sealed Balch tubes were incubated statically under a light intensity of 60 μE at 31°C.

### Preparation of latex coatings under modified storage conditions

Coatings were prepared from a single culture of *R. palustris* using a separate template mask for each stabilizer combination. After the latex/cell/stabilizer mixture was allowed to coalesce for 24 h, triplicate coatings from each treatment were removed from their respective masks and placed into separate Balch tubes containing PM(NF) medium to assay H_2_ production (argon atmosphere; H_2_ accumulation was measured at 5-day intervals over 20 days). The remaining coatings were removed from the mask, placed in Petri dishes, covered with foil, and stored at 22°C under 60% humidity for 14, 28 or 56 days before assaying for H_2_ production. One set of triplicate coatings was stored at < 5% humidity for 28 days.

### Gas analysis

Headspace gas analysis was performed using an Agilent 6890 gas chromatograph (Agilent Technologies, Santa Clara, CA) equipped with a thermal conductivity detector and a HP-Molseive column (30 m × 0.32 mm × 25 μm). Argon was the carrier gas and the oven, detector and inlet temperature settings were 50°C, 275°C and 105°C, respectively, yielding a RT of 6.02 min for H_2_ and baseline separation of N_2_ and O_2_. H_2_ was quantified by comparison of peak areas to standard curves constructed from known amounts of H_2_ gas (Gosse *et al*., 2007).

### CTC staining and confocal microscopy

The respiratory activity of *R. palustris* CGA009 cells in latex coatings was assessed under anaerobic conditions using 5-cyano-2,3-ditolyl tetrazolium chloride dye (CTC; Sigma-Aldrich, St. Louis, MO). CTC is internalized and reduced by actively respiring cells to a fluorescent CTC-formazan that can be detected by epifluorescence microscopy (Rodriguez *et al*., 1992; Yu and McFeters, 1994).

Coatings of *R. palustris* CGA009, prepared with a latex/sucrose/glycerol formulation, were dried at 60% humidity for 3 h and then stored in the dark at either 60% or < 5% humidity for 2, 14, 28 or 56 days. Additionally, coatings, prepared with sucrose or trehalose, were stored in septa-sealed Balch tubes containing drierite for 8−12 weeks under aerobic (a needle attached to a 0.2-micron syringe filter was passed through the septum) or anaerobic conditions (tubes were sealed and flushed extensively with argon; low O_2_ concentrations were confirmed by GC-TCD). Following the storage period, coatings were hydrated in PM(NF) medium and pre-incubated in sealed Balch tubes under an argon headspace for 4 days, after which CTC dye was injected to a final concentration of 4 mM. Coatings were incubated anaerobically with CTC in the dark for 1 h with constant shaking (100 r.p.m.) at 31°C. Heat-treated (85°C for 0.5 h) and unstained latex coatings were also examined as controls.

A confocal laser-scanning microscope (LSM 510 Meta, Carl Zeiss Microimaging) equipped with a HeNe1 laser was used to view the CTC-stained latex coatings. Images were collected at an excitation wavelength of 543 nm, a master gain of 633 V and with an alpha Plan-Fluor 100×/1.45 oil objective. The confocal microscope facilitated viewing the cells at different depths (z-axis) within the latex coating matrix. Fluorescent spots were enumerated for three randomly selected fields of view for each plane of analysis (coating depth) using the AlphaEase FC counting software (AlphaImager 3400; Alpha Innotech Corporation, San Leandro, CA).

## Conflict of interest

None declared.

## Supporting information

**Fig. S1.** Illustration of the *R. palustris latex* coating method.
