# Supplementary material for: Preservation of H2 production activity in nanoporous latex coatings of Rhodopseudomonas palustris CGA009 during dry storage at ambient temperatures
Source: Microb Biotechnol. 2013 Jul 1;6(5):515–25. doi: 10.1111/1751-7915.12032 (PMC3918154; doi:10.1111/1751-7915.12032)
Supplement: Supplementary file 1 — Fig. S1. Illustration of the R. palustris latex coating method. [file mbt0006-0515-sd1.pdf]

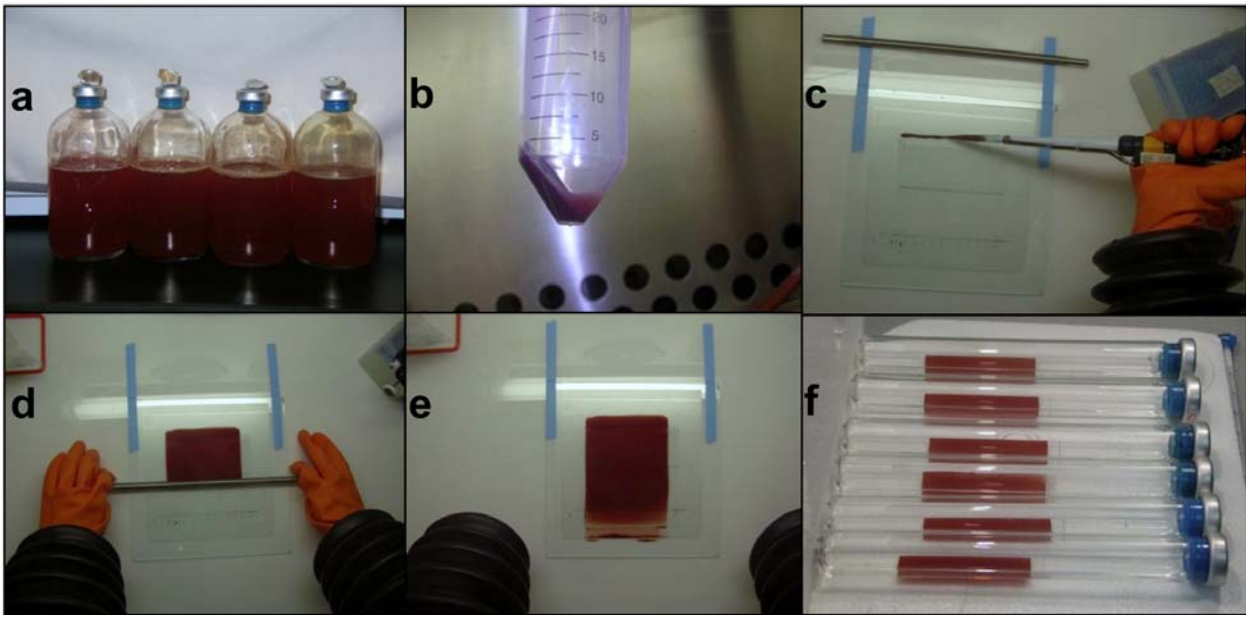

Supporting Figure 1. Illustration of the *R. palustris* latex coating method. (A) Cultures of *R. palustris* grown anaerobically in serum vials; (B) Pellet of *R. palustris* cells from the 4 serum vials; (C) Application of cell/latex/osmotic stabilizer mixture across the top of a pre-cut polyester mask in a humidity-controlled chamber; (D) Spreading cell/latex mixture using a Mayer rod; (E) Drying coatings in the pre-cut mask under air with humidity control; (F) Coating strips hydrated in PM(NF) medium under argon headspace in Balch tubes
